# The effect of air pollutants on COPD-hospitalized patients in Lanzhou, China (2015–2019)

**DOI:** 10.3389/fpubh.2024.1399662

**Published:** 2024-09-19

**Authors:** Limei Jin, Shuya Fang, Yaxing Nan, Jihong Hu, Hua Jin

**Affiliations:** ^1^School of Public Health, Gansu University of Chinese Medicine, Lanzhou, China; ^2^Wenling Meteorological Bureau, Wenling, China; ^3^School of Health Management, Gansu University of Chinese Medicine, Lanzhou, China; ^4^Key Laboratory of Dunhuang Medicine, Ministry of Education, Lanzhou, China; ^5^Clinical College of Chinese Medicine, Gansu University of Chinese Medicine, Lanzhou, China

**Keywords:** air pollutants, COPD, ecological study, distributed lag non-linear model, Lanzhou

## Abstract

**Background:**

Lanzhou is the largest heavy industrial city in northwest China and it is a typical geographical valley-like city. However, there are few studies on the relationship between air pollutants and COPD, and their respective sample sizes are small, resulting in inconsistent results. The aim of this study is to analyze the effects of air pollutants on COPD hospitalizations in Lanzhou, China.

**Methods:**

An ecological time series study with distributed lag non-linear model (DLNM) was used for analysis. Daily COPD hospitalization data in Lanzhou from 1 January 2015 to 31 December 2019 were collected from 25 hospitals, as well as air pollutant data and meteorological data.

**Results:**

A total of 18,275 COPD hospitalizations were enrolled. For 10 μg/m^3^ increase in PM_2.5_, PM_10_, SO_2_, NO_2_, and 1 mg/m^3^ increase in CO at lag 07 day, the RR95%CI of COPD hospitalizations were 1.048 (1.030, 1.067), 1.008 (1.004, 1.013), 1.091 (1.048, 1.135), 1.043 (1.018, 1.068), and 1.160 (1.084, 1.242), respectively. The exposure–response curves between air pollutants (except O3-8h) and COPD hospitalizations were approximately linear with no thresholds. Female, and the harmful effect of PM on aged <65 years, the effect of gaseous pollutant on those aged ≥65 years, were stronger, particularly in the cold season. Exposure to air pollutants (except O_3-8h_) might increase the risk of COPD hospitalizations. O_3-8h_ has a weak and unstable effect on COPD.

**Conclusion:**

Exposure to air pollutants (except O_3-8h_) increases the risk of COPD hospitalizations. O_3-8h_ has a weak and unstable effect on COPD hospital admissions. The harmful effect of gaseous pollutants (except O_3-8h_) on COPD-hospitalized patients was stronger than that of PM.

## Introduction

WHO data showed that 99% of the global population breathes air that exceeds WHO guideline limits and contains high levels of pollutants, with low-and middle-income countries (LMICs) suffering from the highest exposures. In addition, exposure to air pollutants will have many adverse effects on health, such as the increase in morbidity and mortality of some diseases, including chronic non-communicable diseases (NCDs) and infectious diseases in various systems of the human body (1–3). The Global Burden of Disease (GBD) study showed that air pollution is the leading level 2 risk factor for GBD among all environmental and occupational risks and the fourth leading cause of global attributable death for humans (4). China’s rapid economic development over the past few decades has resulted in a significant increase in air pollutant emissions, and despite continuous improvement in air quality as a result of a series of strict control policies, such as the Action Plan for Air Pollution Prevention and Control and the Three-year Action Plan for Winning the Blue Sky Battle, air pollution remains an important public health threat in China. In 2019, air pollution caused approximately 1.85 million premature deaths in China (4).

The GBD study also showed that NCDs had the largest risk-attributable burden in 2017, and chronic obstructive pulmonary disease (COPD) is one of the top five leading causes of risk-attributable burden, with 633,000 deaths (5). In 2019, 212.3 million prevalent cases of COPD were reported globally, with COPD accounting for 3.3 million deaths (6), and with more than three-fourths of cases in LMICs. Research also shows that the morbidity and mortality due to chronic respiratory disease continue to increase, and the increase is driven primarily by the growth of COPD (7). Therefore, COPD is an increasingly important cause of morbidity, disability, and mortality worldwide; it remains a major public health problem, especially in LMICs (8). Current research focuses on the impact of air pollution on COPD. For example, most recent estimates suggest that 50% of the total attributable risk of COPD may be related to air pollution (9), while a study in Lahore city, Pakistan, showed that PM_2.5_ contributes 31.41% attributable proportion (AP) to COPD in adults (10). A cohort study based on the UK Biobank of 265,506 adults showed that air pollutants were associated with the incidence and progression trajectory of COPD (11). A study based on the pooled cohort from existing cohorts in Sweden, Denmark, Germany, the Netherlands, Austria, France, and Italy found significant positive associations between PM_2.5_, NO_2_, and COPD hospital admissions (12). A study (13) conducted in Jinan, China, showed that the daily COPD hospital admissions increased by 2.36% (95%CI: 0.13–4.65%) and 2.39% (95%CI: 0.19–4.65%) for every 10 μg/m^3^ increase in NO_2_ and SO_2_ concentrations, respectively. However, no statistically significant associations were found between COPD and PM_2.5_, PM_10_, CO, and O_3_.

Lanzhou is one of the largest heavy industry cities in northwest China and a typical mountain valley city (14). At the same time, with the rapid development of urbanization, industrialization, and economy in Lanzhou, as well as a significant increase in car ownership and other factors, haze, sand, and dust pollution events still frequently occur in Lanzhou; moreover, the concentration of air pollutants in Lanzhou still remains higher than the national average level (15). Some studies (16, 17) have also analyzed the relationship between air pollutants and COPD in Lanzhou. However, specifically, Bao’s study (16) only analyzed the association between particulate matter (PM_2.5_ and PM_10_) and hospital outpatient visits for COPD. Furthermore, Dong’s (17) study analyzed the association between six ambient air pollutants and outpatient visits for acute exacerbation of COPD. In addition, both studies only collected COPD data from three hospitals, resulting in inadequate representation and inconsistent results. Therefore, in this study, we collected COPD hospitalization data from 25 hospitals in Lanzhou and explored the relationship between air pollution and COPD in Lanzhou by a distributed lag non-linear model (DLNM).

## Materials and methods

### Study area

Lanzhou is located in the interior of northwest China; it is the capital city of the Gansu Province. The city has four main districts (Chengguan, Qilihe, Xigu, Anning). The geographical location, urban area, population, and climate are reported in our previous articles (18, 19).

### Data sources

**Figure 1:**
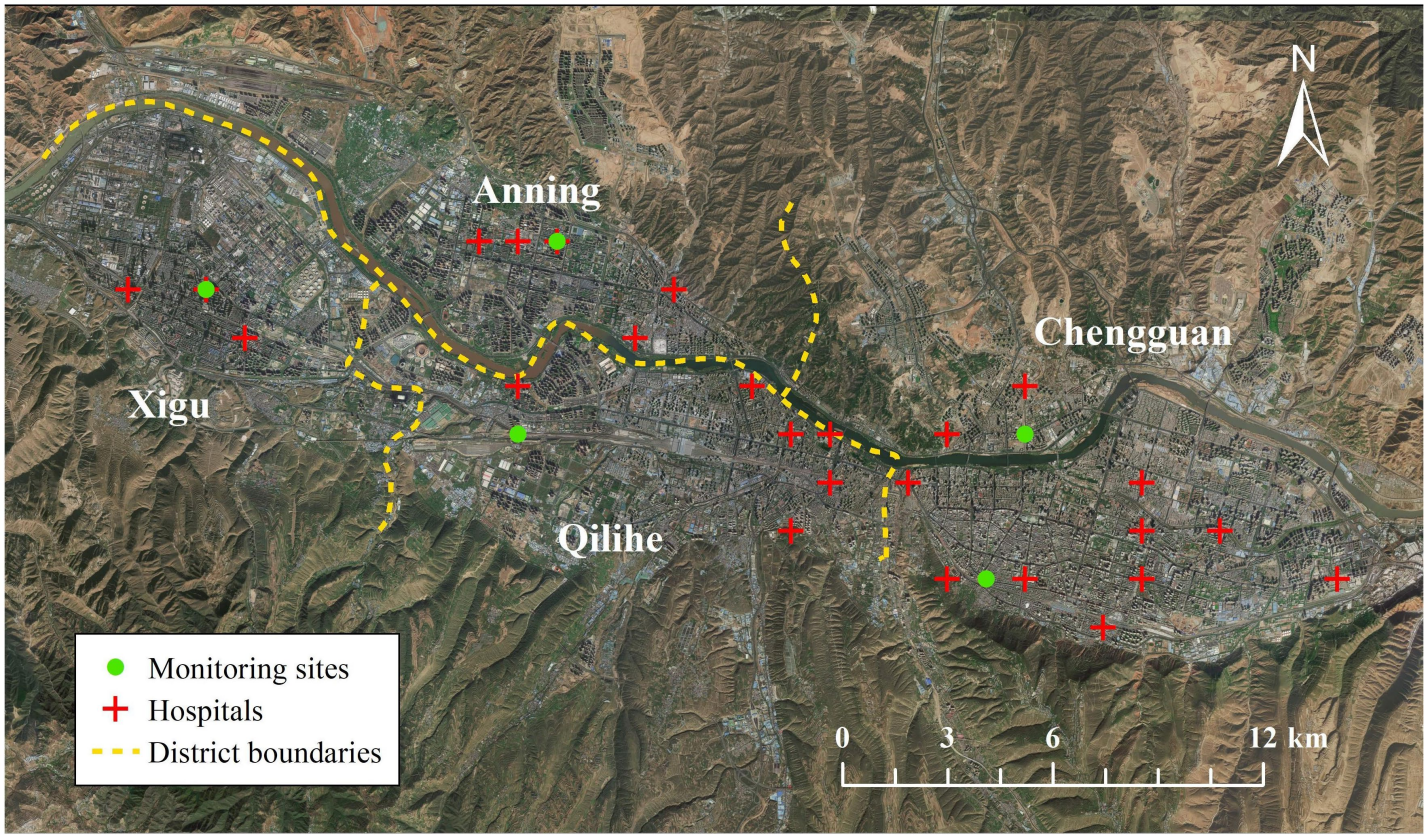
The locations of hospitals and air pollutants monitoring sites.

In 2015, most hospitals in Lanzhou City had a complete electronic medical record system. However, in 2020, upon the emergence of the novel coronavirus epidemic, hospitalization data were abnormal. Hence, data on daily COPD hospital admissions from 1 January 2015 to 31 December 2019 were collected from 25 hospitals, which have complete electronic medical record systems. Hospitals’ locations are shown in Figure 1. Data included the following: age, gender, residential address, date of hospital admission, and principal diagnosis. Inclusion criteria include: Patients diagnosed with COPD by the attending physician, diagnosed according to the Chronic Obstructive Pulmonary Disease (GOLD) criteria, with risk factors and/or clinical symptoms, and lung function indicating persistent airflow limitation (FEV1/FVC < 0.70 after bronchodilator use). The International Classification of Diseases, 10th Revision (ICD-10) codes is J44. Exclusion criteria include: (1) patients with a residential address outside of the urban districts in Lanzhou and (2) incomplete records. A total of 18,275 eligible subjects, aged 28 to 104, were included.

Data on hourly air pollution from 1 January 2015 to 31 December 2019 were collected from five national environmental monitoring stations in the city of Lanzhou, including PM_2.5_, PM_10_, SO_2_, NO_2_, CO, and O_3_. Figure 1 shows the locations of the five monitoring stations for air pollutants. Our previous studies reported the calculated daily 24-h mean concentrations of PM_2.5_, PM_10_, SO_2_, NO_2_, CO, and the maximum daily 8-h moving average of O_3_ (O_3_8h) (18, 19).

The daily average temperature (T, °C) and daily average relative humidity (RH, %) were obtained from the China Meteorological Data Service Center.^1^

### Statistical analysis

According to previous studies (10, 20), the association between air pollution and COPD outcomes is potentially non-linear and lagged, so time series analysis of the effects of air pollutants on COPD using the distributed lag non-linear model (DLNM), which can describe both non-linear exposure–response curves and lag–response relationships (21).

The constructed model is shown below: Log[*E*(*Yt*)] = *α* + *βX*t*,l* + *ns*(*Time*, *df* = 7/year) + *ns*(*Temt*, *df* = 3) + *ns*(*RHt*, *df* = 3) + *Dow* + *Hol*

*t:* the time observed (days); *E*(*Yt*): the expected mean of number of COPD hospital admissions at *t* day; *Xt*,*l*: cross-basis matrix obtained by series of transformations for daily mean concentration of a certain air pollutant, *l:* the maximum lag days; *β:* the coefficient of the matrix; *ns*: the function of natural cubic spline, the *ns* was used to control the non-linear confounders such as long-term and seasonal trends of the daily COPD hospital admissions, the average temperature, and the average relative humidity; *Time*: the time variable, *df*: the degree of freedom; *Temt*: the average temperature on day *t*; *RHt*: the average relative humidity on day *t; Dow*: the day of the week effect, and *Hol*: the public holiday effect.

We analyzed the relationship between air pollutants and the total COPD hospital admissions population by using DLNM, and also stratification was analyzed by gender (male and female), age (<65 years and ≥ 65 years old), and season (warm season and cold season). The cold season is from November to March (the heating season of Lanzhou City), and the warm season is from April to October (the non-heating season). Moreover, to test the robustness of the model, the lag day with the largest estimated effect in the single-pollutant model was chosen to perform the sensitivity analysis. (1) The two-pollutant models, except PM_2.5_ and PM_10_. (2) The *df* of the *ns* function of *Time* was changed from 6 to 10. The relative risk (RR) and corresponding 95% confidence interval (CI) were calculated to estimate the effect of each 10 μg/m^3^ (CO, 1 mg/m^3^) increase for each air pollutant on COPD. The basic statistical analysis was conducted using SPSS software, version 22.0. The regression analysis of DLNM was conducted using R software version 3.6.3, with its “dlnm” package. Details of the statistical analysis are reported in our previous articles (18, 19).

## Results

### Statistical description

Tables 1, 2 show the descriptive statistics on COPD hospital admissions and the air pollutants in Lanzhou City during 2015–2019. A total of 18,275 COPD hospital admissions were collected, including 11,850 males and 6,425 females, 3,516 cases aged <65 years, and 14,759 cases aged ≥65 years. The average daily hospitalization of COPD in the cold season was 11.1 ± 6.9 cases/day, which was much higher than in the warm season (9.0 ± 5.8 cases/day). Meanwhile, the average concentrations of PM_2.5_, PM_10_, SO_2_, NO_2_, and CO in cold seasons were also higher than in the warm seasons (all *p-*values<0.0001). Whereas, there was no statistical difference in the average concentrations of O_3-8h_ between the cold and warm seasons (*p* = 0.894).

**Table 1:** Descriptive statistics of hospital admissions for COPD in Lanzhou City, 2015–2019.

	Daily hospital admissions						Total of hospital admissions
	Mean ± SD	Min	*P_25_*	*P_50_*	*P_75_*	Max	
All	9.9 ± 6.4	0	5	9	22	41	18,275
Gender							
Male	6.4 ± 4.4	0	3	6	14	26	11,850
Female	3.5 ± 2.8	0	1	3	9	16	6,425
Age							
<65 years	1.9 ± 1.8	0	0	1	6	9	3,516
≥65 years	8.0 ± 5.3	0	4	7	18	37	14,759
Season							
Cold	11.1 ± 6.9	0	6	10	15	41	8,552
Warm	9.0 ± 5.8	0	4	8	12	35	9,723

**Table 2:** Descriptive statistics of air pollutants in Lanzhou City, 2015–2019.

Air pollutants	Mean	*SD*	Min	*P_25_*	*P_50_*	*P_75_*	Max
PM_2.5_ (μg/m^3^)	48.45	26.26	8.58	31.24	41.99	57.78	276.50
Warm	37.61	18.39	8.58	26.94	34.96	44.39	276.50
Cold	63.94	28.02	12.73	43.05	57.66	79.04	233.14
PM_10_ (μg/m^3^)	118.05	89.66	16.08	71.77	100.38	139.72	1484.54
Warm	97.44	74.13	16.08	60.77	84.92	112.68	1242.27
Cold	147.53	101.05	36.10	94.62	128.91	169.43	1484.54
SO_2_ (μg/m^3^)	19.96	13.70	3.54	9.91	15.33	26.46	80.79
Warm	12.25	5.80	3.54	7.91	10.96	15.15	41.82
Cold	30.98	14.20	7.27	19.99	28.81	40.40	80.79
NO_2_ (μg/m^3^)	49.39	18.18	12.58	37.58	47.31	56.82	145.53
Warm	43.97	12.67	12.58	34.80	44.77	52.04	90.53
Cold	57.15	21.72	13.21	42.23	52.79	68.66	145.53
O_3-8h_ (μg/m^3^)	47.95	22.19	7.80	30.62	44.71	62.50	126.90
Warm	48.00	22.42	7.80	30.70	44.44	62.85	123.20
Cold	47.87	21.87	10.13	30.60	45.06	62.35	126.90
CO (mg/m^3^)	1.21	0.72	0.30	0.73	0.96	1.41	4.64
Warm	0.84	0.26	0.30	0.66	0.81	0.98	2.02
Cold	1.75	0.83	0.36	1.07	1.56	2.33	4.64

The Spearman rank correlation (*rs*) between daily COPD admissions, air pollutants, and meteorological factors is shown in Table 3. Daily COPD admissions were significantly positively correlated with daily PM, SO_2_, NO_2_, and CO concentrations, but significantly negatively correlated with T and RH. Except for SO_2_ and O_3-8h_, there were significant correlations among air pollutant concentrations, with the highest *rs* between PM_2.5_ and PM_10_ (0.876) being the highest. Air pollutants other than O_3-8h_ were negatively and significantly correlated with T, as well as RH.

**Table 3:** The coefficient of spearman rank correlation (*r_s_*) between daily COPD admissions, air pollutants and meteorological data in Lanzhou, 2015–2019.

	COPD	PM_2.5_	PM_10_	SO_2_	NO_2_	O_3-8h_	CO	T	RH
COPD	1.000	0.115^*^	0.090^*^	0.166^*^	0.170^*^	−0.024	0.084^*^	−0.215^*^	−0.053^Δ^
PM_2.5_		1.000^*^	0.876^*^	0.649^*^	0.515^*^	0.208^*^	0.698^*^	−0.547^*^	−0.156^*^
PM_10_			1.000^*^	0.580^*^	0.460^*^	0.203^*^	0.548^*^	−0.385^*^	−0.390^*^
SO_2_				1.000^*^	0.582^*^	0.011	0.777^*^	−0.668^*^	−0.253^*^
NO_2_					1.000^*^	0.069^Δ^	0.635^*^	−0.292^*^	−0.179^*^
O_3-8h_						1.000^*^	0.088^*^	−0.008	−0.031
CO							1.000^*^	−0.559^*^	−0.034
T								1.000^*^	−0.031
RH									1.000^*^

T, temperature; RH, relative humidity; ^*^p < 0.0001; ^Δ^ *p* < 0.05.

### Effects of air pollutants on COPD admissions analyzed by DLNM

**Figure 2:**
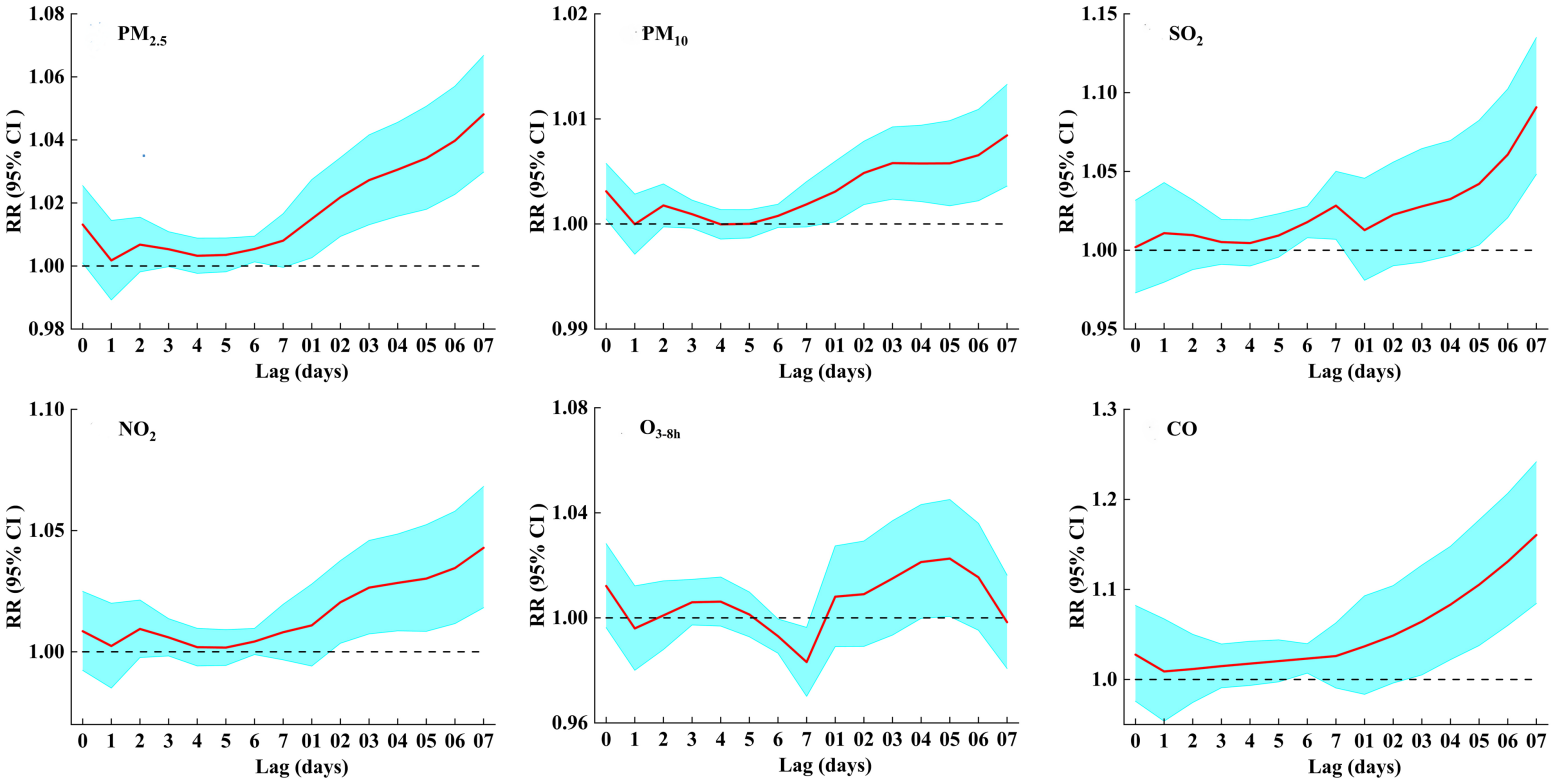
RRs (95% CI) of COPD hospital admissions associated with air pollutants (per 10 μg/m^3^ increase and 1 mg/m^3^ increase in CO) at various lags.

Figure 2 and Supplementary Table S1 show the lag-response plots of the association between air pollutants and COPD hospital admissions. In addition to O_3-8h_, all air pollutants had a significant positive correlation with COPD hospital admissions. The greatest effect of air pollutants on COPD admissions was found at lag07. For 10 μg/m^3^ increase in PM_2.5_, PM_10_, SO_2_, NO_2_, and 1 mg/m^3^ increase in CO at lag 7 day, the RR95%CI of COPD admissions were 1.048 (1.030, 1.067), 1.008 (1.004, 1.013), 1.091 (1.048, 1.135), 1.043 (1.018, 1.068), and 1.160 (1.084, 1.242). However, for 10 μg/m^3^ increase in O_3-8h_, the RR95%CI was 0.983 (0.970, 0.996) at lag 7, and 1.023 (1.001, 1.045) at lag 5. This was obvious that air pollutants (except O_3-8h_) may had an adverse effect on COPD hospital admissions, and the adverse effect of PM_2.5_ was higher than that of PM_10_, with CO having the strongest harmful effect.

**Figure 3:**
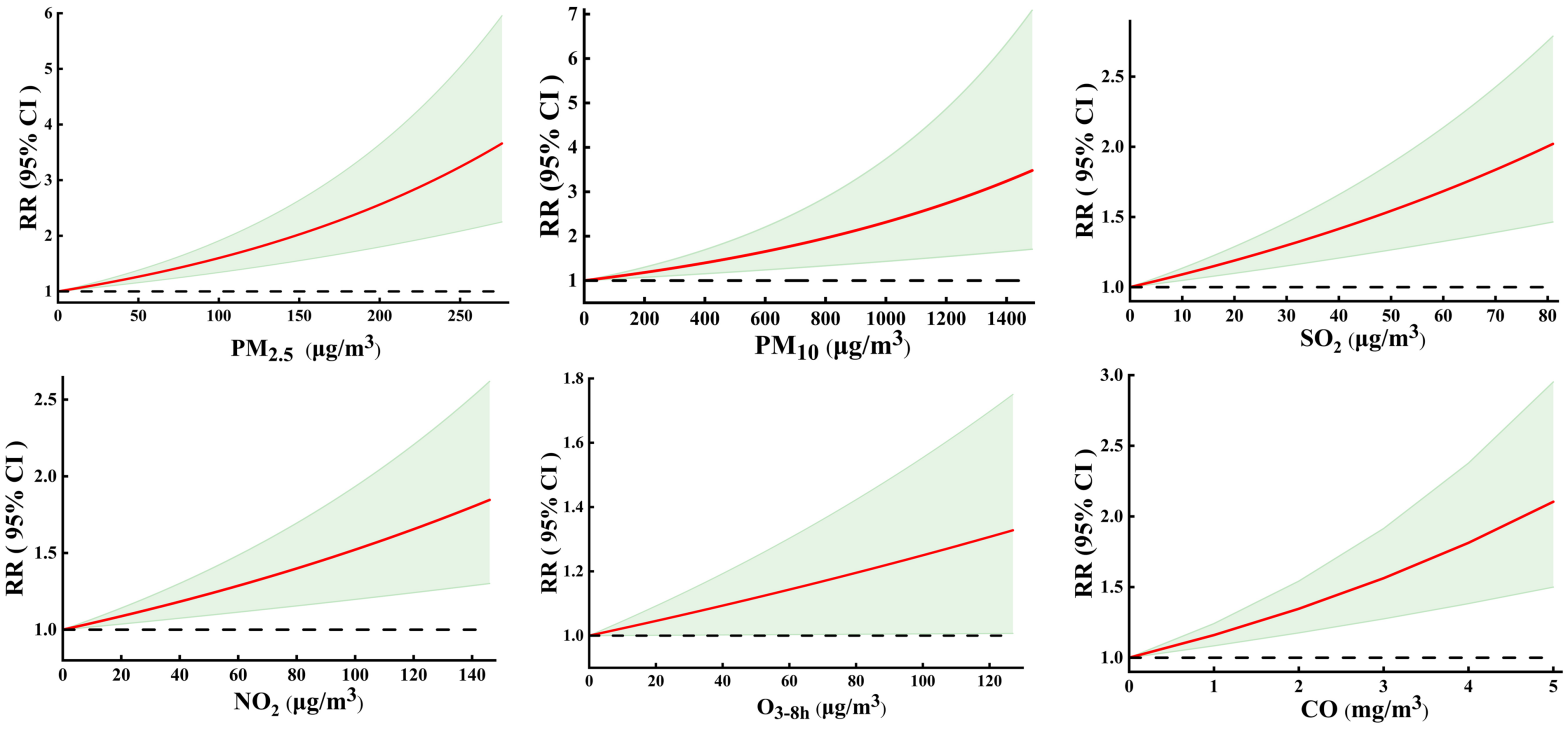
Exposure-response curves between air pollutants and COPD hospital admissions at lag07 (O_3-8h_ at lag 05 day).

Figure 3 shows the exposure–response curves of the effects of air pollutants on COPD hospital admissions. Based on the results of Figure 2 and Supplementary Table S1, we focused on COPD admissions related to air pollutants at lag 7 day (O_3-8h_ at lag 5 day). The six exposure–response curves were approximately linear, with the curves of PM_2.5_, PM_10_, SO_2_, NO_2_, and CO showing a significant positive relationship and no thresholds, but the exposure–response curve of O_3-8h_ was not statistically significant.

### Stratified analysis

**Figure 4:**
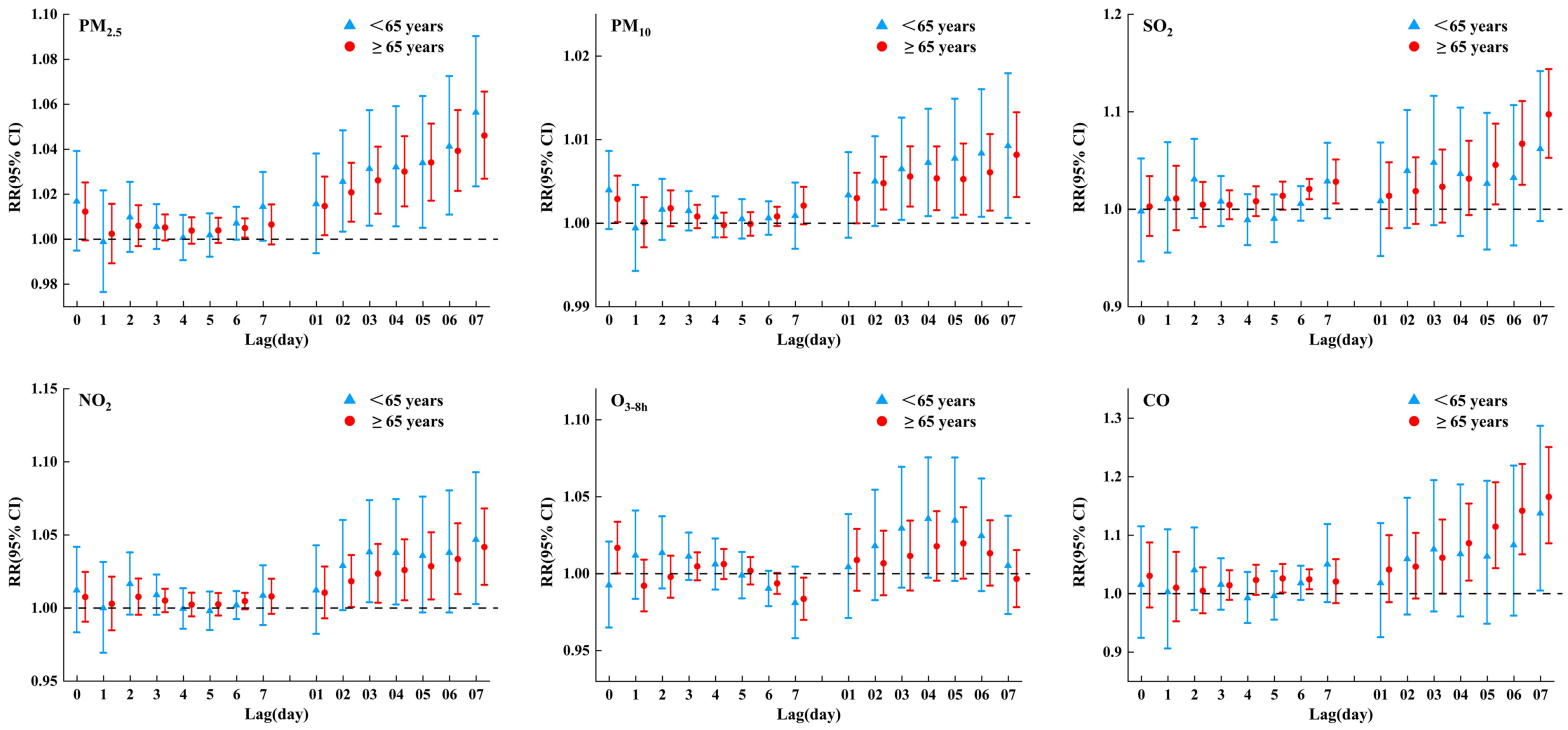
RRs (95% CI) of COPD hospital admissions associated with air pollutants (per 10 μg/m^3^ increase and 1 mg/m^3^ increase in CO) at various lags stratified by age in single pollutant model.

Figure 4 shows the RRs (95% CI) of COPD admissions associated with air pollutants stratified by age. In those aged <65 years, only PM_2.5_, PM_10_, NO_2_, and CO were significantly and positively associated with COPD hospital admissions. In those aged ≥65 years, COPD hospital admissions were negatively correlated with O_3-8h_ but positively with other air pollutants. Simultaneously, the harmful effect of particulate matter on those aged <65 years was slightly higher than those aged ≥65 years; however, the effect of the gaseous pollutants on those aged <65 years was slightly lower than those aged ≥65 years.

**Figure 5:**
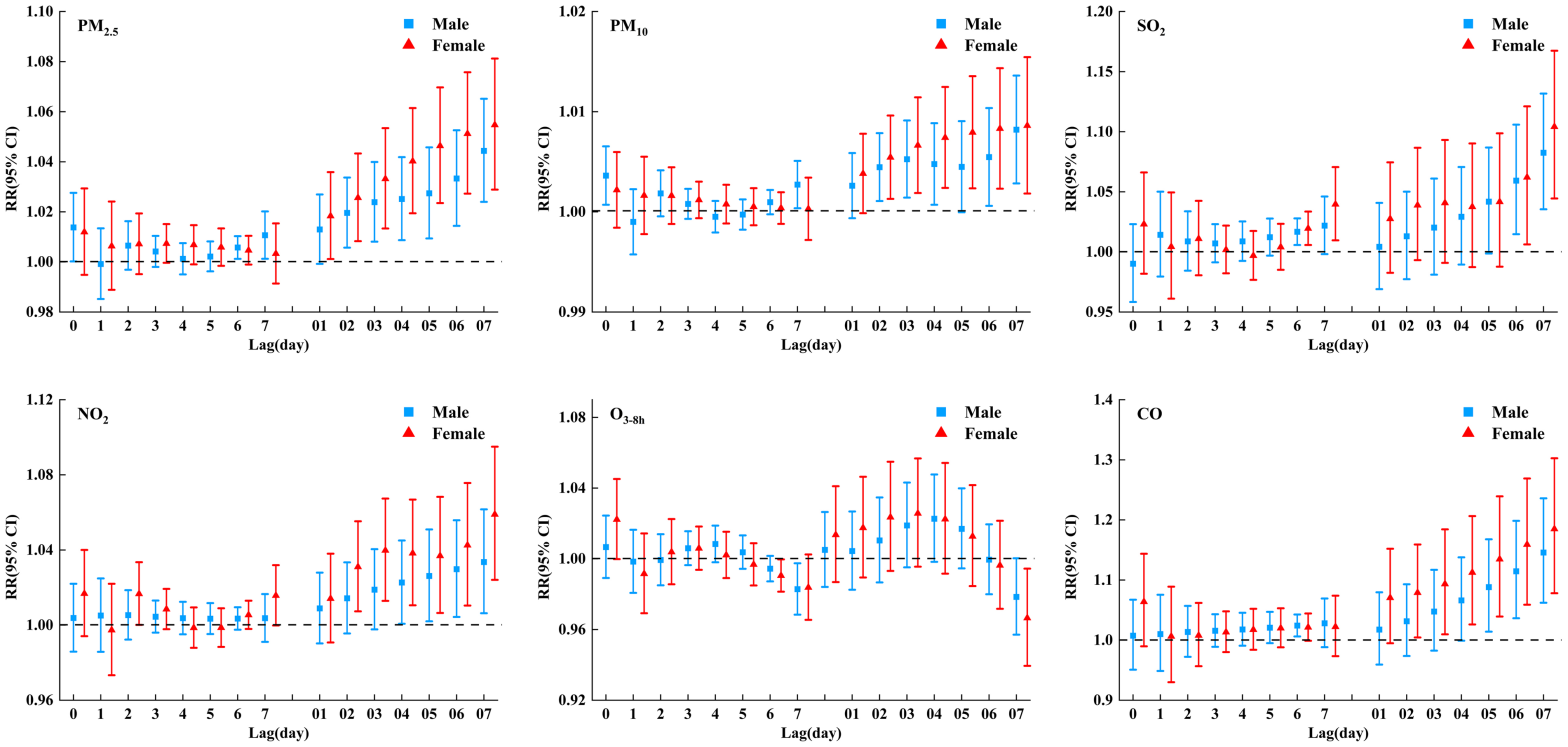
RRs (95% CI) of COPD hospital admissions associated with air pollutants (per 10 μg/m^3^ increase and 1 mg/m^3^ increase in CO) at various lags stratified by gender in single pollutant model.

Figure 5 shows a gender-stratified analysis of the effects of air pollutants on COPD. As shown in the figure, all air pollutants had an impact on COPD hospital admissions of different genders. In addition to O_3-8h_, there was a weakly negative correlation between O_3-8h_ and COPD hospital admissions of different genders, and other pollutants were positively correlated with COPD of different genders. That is, air pollutants (except O_3-8h_) have adverse effects on COPD of different genders, and the impact on females was slightly higher than that on males.

**Figure 6:**
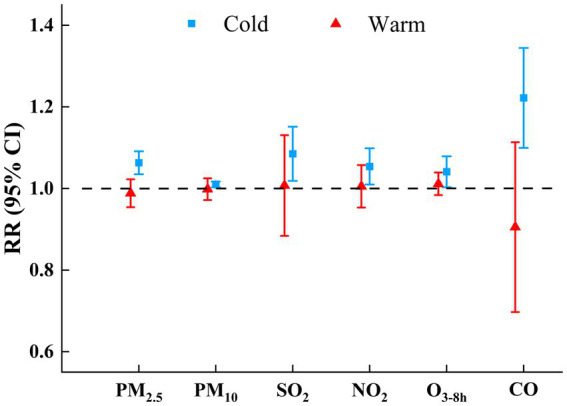
RRs (95% CIs) of COPD hospital admissions with an increase of 10μg/m^3^ in air pollutants (1 mg/m^3^ in CO) by season in single pollutant model.

Stratified analyses according to season (Figure 6; Supplementary Tables S2, S3) revealed that air pollutants (except O_3-8h_) had statistically adverse effects on COPD hospital admissions during the cold season (heating period), while no statistical effects were observed in the warm season. COPD had a significant positive correlation with O_3-8h_ at lag 3–4 days, but a negative correlation with O_3-8h_ at lag 6–7 days during the cold season, and there was no correlation in the warm season. In conclusion, the influence of air pollutants on COPD in cold seasons was greater than that in warm seasons.

### Sensitivity analysis

**Figure 7:**
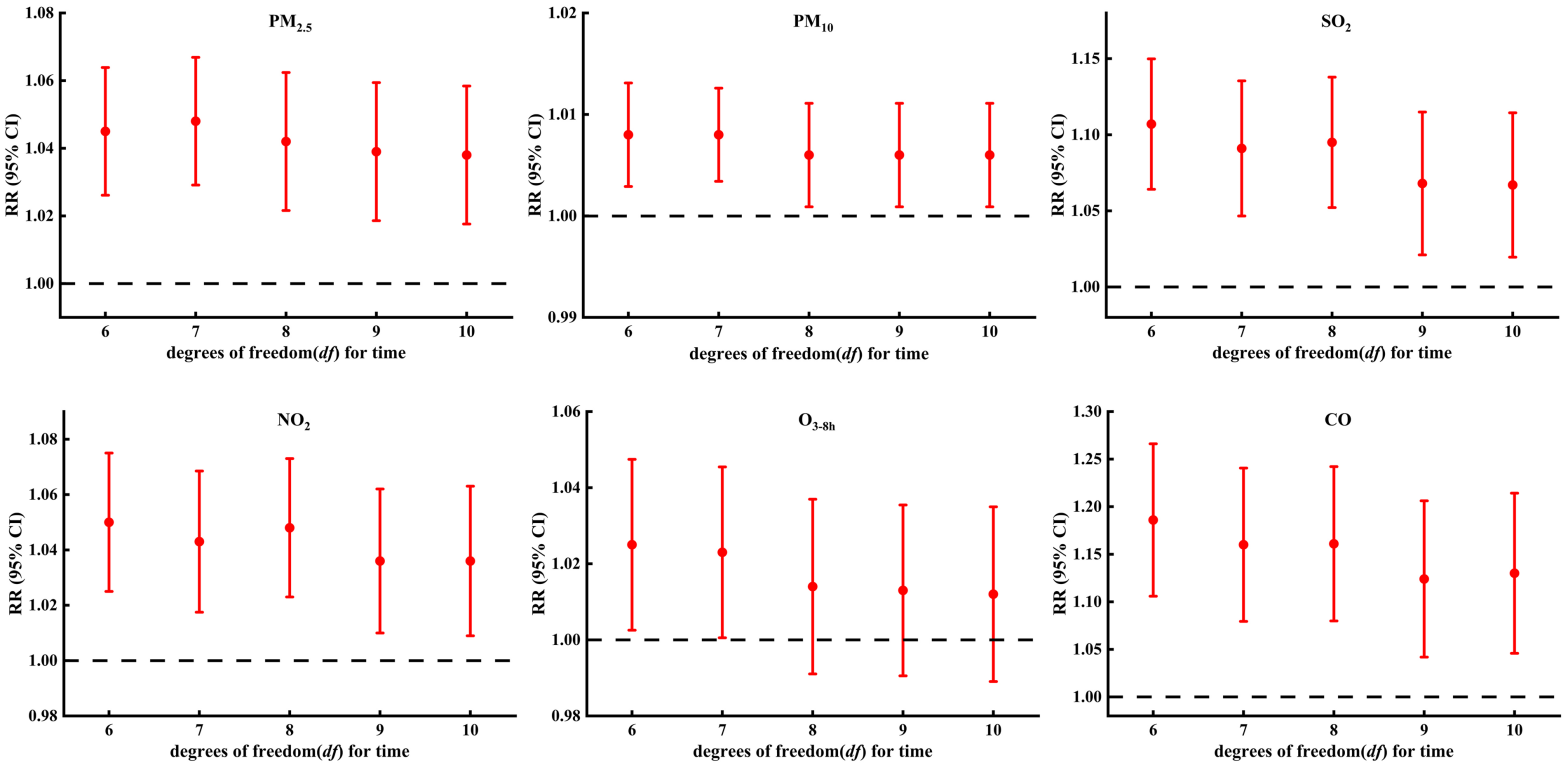
Sensitivity analysis of association between COPD hospital admissions and air pollutants: controlling for different degrees of freedom (df) for time.

**Figure 8:**
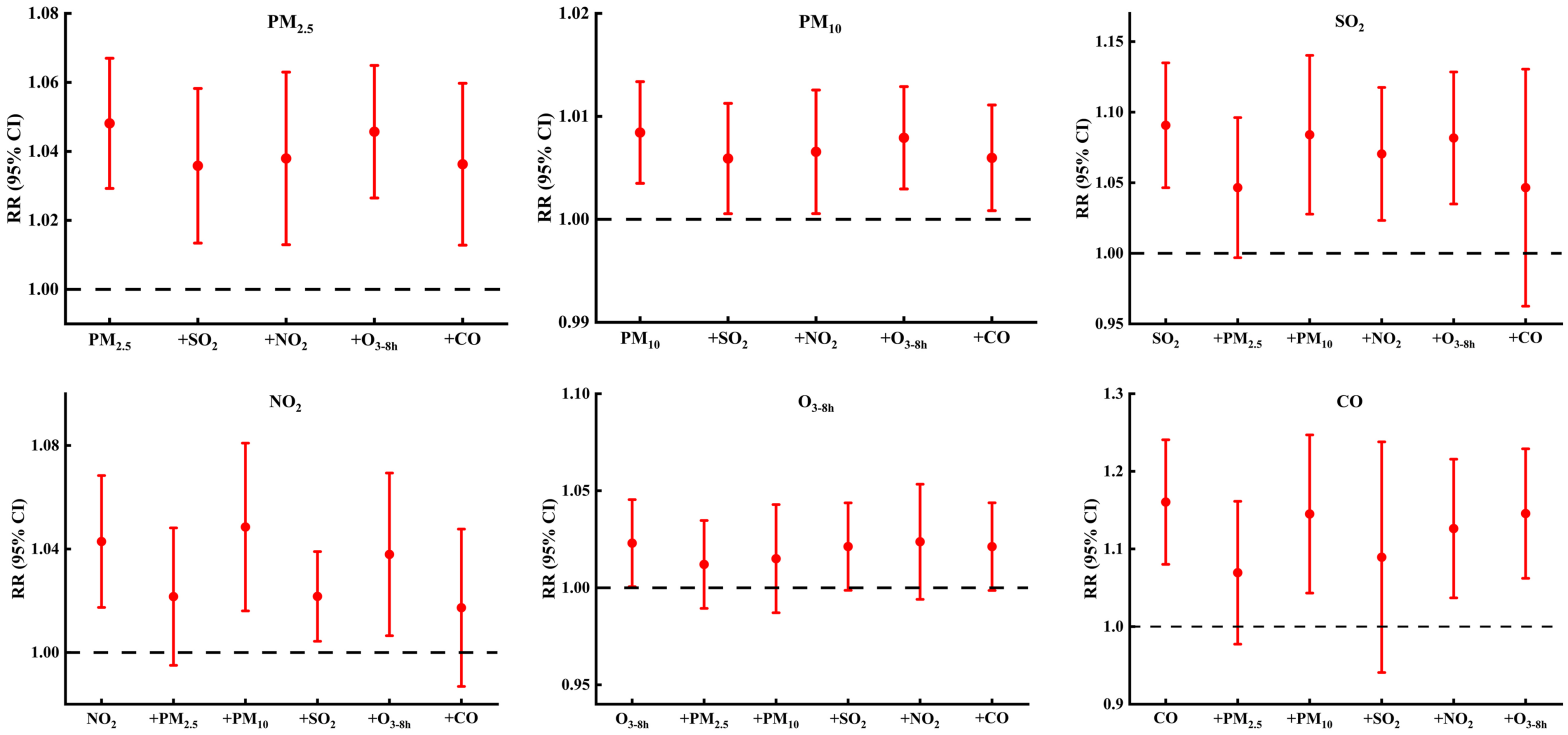
RR (95% CIs) of COPD hospital admissions with an increase of 10μg/m^3^ in air pollutants (1 mg/m3 in CO) from single-pollutant and two-pollutant models.

After further adjustment of the *df* for calendar time (6–10 *df* per year) (Figure 7; Supplementary Table S4), the estimated effects of air pollutants (except O_3-8h_) on COPD hospital admissions changed slightly but the effects were still significant. Two-pollutant models (Figure 8; Supplementary Table S5) showed that, for PM, although the estimated effects changed after the introduction of other air pollutants, the change was small, and the impact on COPD hospital admissions was still significant. For SO_2_ and NO_2_, after further adjustment of other air pollutants (except PM_2.5_ and CO), the relationships between SO_2_, NO_2_, and COPD hospital admissions were consistent with the main model, but the estimated effects changed slightly. For CO, the effects on COPD hospital admissions were still significant after the introduction of other air pollutants (except PM_2.5_ and SO_2_). The main model (single-pollutant model) was statistically significant, whereas some two-pollutant models were insignificant; this may be related to the co-linearity between different air pollutants (22), such as the high *rs* (Table 3) between PM_2.5_ and SO_2_ (*rs* = 0.649), PM_2.5_ and CO (*rs* = 0.698), and SO_2_ and CO (*rs* = 0.777). For O_3-8h_, after further adjustment of other air pollutants, the weakly positive correlation between O_3-8h_ and COPD hospital admissions was insignificant. Overall, the above results suggested that DLNM was robust, and the results between COPD hospital admissions and air pollutants (except O_3-8h_) were stable.

## Discussion

COPD is a global health emergency that affects people from all countries, socioeconomic classes, and age groups, although it disproportionately affects poor and disadvantaged people (7). In this ecological study, we used the COPD hospital admissions data from 25 hospitals and the air pollutants concentration monitoring data in Lanzhou City, and we used the DNLM method to analyze the impact of air pollutants on COPD. We found a statistically significant, stable, and negative connection between air pollution (except O_3-8h_) and the risk of COPD inpatients, where lag effects were identified. For 10 μg/m^3^ increase in PM_2.5_, PM_10_, SO_2_, NO_2_, and 1 mg/m^3^ increase in CO at lag 7 day, the RR95%CI of COPD admissions were 1.048 (1.030, 1.067), 1.008 (1.004, 1.013), 1.091 (1.048, 1.135), 1.043 (1.018, 1.068), and 1.160 (1.084, 1.242), respectively. We also found that the exposure–response curves for pollutants were linear and without obvious thresholds. Moreover, particulate matter had more adverse effects on females and those aged <65 years, while gaseous pollutants (except O_3-8h_) had more adverse effects on females and those aged ≥65 years. Furthermore, the adverse effects of air pollutants in the cold season were greater than those in the warm seasons. Our results establish the hypothesis that air pollutants are a risk factor for COPD and provide baseline data for COPD prevention and air pollution control in Lanzhou.

### PM

Previous studies reported a similar negative correlation between PM and COPD. Such as, an ecological study (10) in Pakistan, found that PM_2.5_ contributes 31.41% attributable-proportion to COPD in adults age 30+. Another nationwide ecological study in Korea (23), that used generalized additive models, found significant associations between COPD (emergency department visits or hospital admissions) and PM; the RR95%CI for COPD for every 10 μg/m^3^ increase in PM_2.5_ and PM_10_ were 1.013 (1.004, 1.022) and 1.014 (1.008, 1.020), respectively. A study in Poland, using DLNM as in this paper (24), also found that the proportions (95%CI) of COPD hospitalizations attributable to air pollution were 7.61% (1.27, 13.49%) for PM_2.5_ and 9.08% (3.10, 15.08%) for PM_10_. An ecological study in Guangdong, China showed that declined attributable hospital admissions for COPD may be associated with the reduction in PM concentrations. Other studies with different methods in different regions have found similar results, such as a cohort study in the UK (11) and in southern China (25), which were based on 265,506 and 580,757 adults, respectively, and found that PM exposure could affect the progression from free of chronic lung disease to incident COPD morbidity and death. A case-crossover study (26) showed that short-term PM_2.5_ exposure was associated with increased odds of exacerbations in Canadians with COPD, further heightening the awareness of non-infectious triggers of COPD exacerbations. A meta-analysis (27) of 37 studies showed that a 10 μg/m^3^ increase in PM_2.5_ was associated with a 1.6–3.4% increase in COPD-related emergency department visits and hospital admissions. A randomized controlled trial (28) in the United States showed that a reduction in PM_2.5_ caused clinically significant improvement in respiratory health for individuals with COPD. It is reported that PM_2.5_-induced cytokine release and oxidative stress are the main mechanisms leading to COPD. Meanwhile, the bacteria, eukaryotes, and viruses in PM may directly cause neutrophilic inflammation or break the microorganism balance, contributing to the development and exacerbation of COPD (29). On the other hand, air pollution may make COPD patients more susceptible to respiratory viral infections, especially influenza virus A (30). Mice experiments also showed that acute PM2.5 exposure increased airway inflammation (31). A human *in vitro* cell culture experiment (32) showed that epithelial remodeling and dendritic cell dysfunction might accelerate inflammation after PM exposure in COPD.

### SO_2_

SO_2_ mainly comes from the combustion of fossil fuels or emissions from industrial activities. The rats experiment has revealed that (33) SO_2_ affects the airway inflammatory and immune responses of the rats and enhances the susceptibility to ovalbumin by aggravating inflammatory responses in the lungs, upregulating proinflammatory cytokine expression, and causing a Th1/Th2 imbalance, which might contribute to the increased risk of respiratory system disease. SO_2_ can penetrate deep into lung tissue and be converted to hydrosulfite, which interacts with sensory receptors, causing respiratory irritation, bronchoconstriction, mucus production, increasing the sensitivity of the respiratory tract to sensitizing substances, etc., and can also induce inflammation and oxidative stress in the whole body, including the lung (34). Prospective cohort data from the Australian Longitudinal Study on Women’s Health also showed that, after controlling for covariates, air pollutants (PM, SO_2_, NO_X_, and CO) modeled individually were significantly associated with the risk of COPD. When modeled together, only SO_2_ remained significantly associated with COPD (35). Evidence from Guangdong, China, showed that a decrease in hospital admissions for COPD is associated with a reduction in concentrations in Guangdong (36). This suggests that improving ambient air quality, a modifiable risk factor, has been shown to reduce COPD hospitalizations. In recent years, with the large-scale deployment of flue gas desulfurization at power plants, strict control of SO_2_ industrial emissions, as well as engine technology innovation and the use of clean fuels and other effective measures in Lanzhou City (37, 38) has been enforced. As a result of this, from 2015 to 2019, SO_2_ concentration in Lanzhou City showed a significant decline trend; furthermore, the average concentration was 19.96 μg/m^3^. Compared with some megacities and heavily polluted cities, such as Arak, Iran (54.83 μg/m^3^) (39), Taiyuan (69.34 μg/m^3^) (40), and Shenyang (55.0 μg/m^3^) (41), China. The level of SO_2_ in Lanzhou is low and meets the national air quality standard level I limit (the average annual standard for SO_2_ is 20 μg/m^3^). However, the harmful effect of SO_2_ on COPD was still found in this study, and the harmful effect was stronger than that of PM. It is suggested that the “low exposure, high risk” effects of SO_2_ on health may need to be considered in the formulation of environmental health policies, and more stringent air pollution intervention policies to protect human respiratory health should be implemented.

### NO_2_

This study found that every 10 μg/m^3^ increase in NO_2_ corresponded to an RR (95%CI) =1.043 (1.018, 1.068) increase in COPD hospital admissions, and the harmful effect was higher than that of PM_10_. Like SO_2_, NO_2_ is also an oxidizing gas. It was reported that exposure to NO_2_ can increase the amount of proinflammatory cytokine, thus aggravating the original inflammatory response of the respiratory system (42). It means that exposure to NO_2_ can further worsen symptoms associated with respiratory infections, such as wheezing and difficulty breathing, forcing infected patients to be hospitalized (43). When NO_2_ reaches the alveoli, the nitric acid and its salts formed with the water on the surface of the alveoli can be stimulated and result in the corrosion of the lung tissue, which results in the destruction of the alveolar epithelium and capillary epithelium. The phagocytotic ability of the alveolar phagocyte are reduced, which enhances the susceptibility of lung tissue to infection (34). The Canadian Cohort Obstructive Lung Disease study (44) showed that NO_2_ exposure was associated with lower lung function, even at low concentrations, especially those at the extremes of dysanapsis. We also found a statistically significant negative connection between NO_2_ and the risk of COPD inpatients. Similar to the results in Canada (26), Warsaw, Cracow, and Tricity, Poland (24), Belluno, Italy (45), Berlin, Germany (46), European Cohorts in the European Study of Cohorts for Air Pollution Effects (ELAPSE) Project (12), a nationwide study in Korea (23). Jinan, China (13), Qingyang, and China (47), all results showed that short-or long-term NO_2_ exposure was associated with increased risks of daily COPD admissions.

### O_3_

O_3_ can cause the muscles in the airways to constrict, trapping air in the alveoli and leading to wheezing and shortness of breath. It can also make the lungs more susceptible to infection (48). However, the results of studies on the relationship between O_3_ and COPD are still inconsistent. This study found that for every 10 μg/m^3^ increase in O_3_, the RR95%CI was 0.976 (0.955, 0.997) at lag 7 and 1.041 (1.005, 1.079) at lag 4. However, the exposure–response curve of the effects of O_3_ on COPD hospital admissions was not statistically significant. Sensitivity analyses revealed that the results between COPD hospital admissions and O_3_ were not stable. That is, O_3_ is not associated with COPD hospital admissions in this study. This is consistent with studies conducted in Berlin, Germany (46), Beijing (49), Shijiazhuang (50), and Jinan (13), China. However, there are also studies showing that exposure to O_3_ can increase (23, 36) or decrease (12, 26, 47) the risk of hospitalization for COPD. An early national multicity study in the United States (51) showed that exposure to O_3_ was negatively associated with COPD admissions on the same day (lag0), but was positively associated with COPD on lag 1. Studies have shown that variability of summer apparent temperature (51) and the use of air conditioning (51, 52) can modify the health effect of O_3_. A recent study on the association between long-term exposure to O_3_ and hospital admissions for four cardiovascular and respiratory outcomes among the Medicare population in the United States (53) suggests that NO_2_ and O_3_ confound each other’s health effects or may be confounded by an unknown or unmeasured variable, and the negative relationship between the NO_2_, O_3_, and health effects is more likely to be affected by unknown or unmeasured confounding factors, while the adverse effects of the two on health are less likely to be affected by confounding factors. So, more studies are needed to ascertain the relationship between O_3_ and COPD hospitalizations.

### CO

This study found that every 1 mg/m^3^ increase in CO corresponded to an RR (95%CI) =1.222(1.108, 1.348) increase in COPD hospital admissions, and the harmful effect was stronger than that of PM. This result was similar to a cohort study in the UK (11), a nationwide study in Korea (23), and studies conducted in Ganzhou (20) and Qingyang City (47) in China. The toxicity of CO is due to its stronger affinity to hemoglobin than oxygen (34), and a review of CO-triggered health effects has shown that CO exposure changed the ability of hemoglobin to transport oxygen to peripheral tissues, induced pulmonary edema, immune cell infiltration, and lung integrity disruption, probably caused respiratory injury, and worsened the symptoms of respiratory diseases (54). A time series study based on clinical randomized controlled trials also showed that exposure to CO reduces lung function (55). In this study, the CO concentration is 1.21 ± 0.72 mg/m^3^, which is below the national air quality standard level I limit of 4 mg/m^3^. This suggests that, like SO_2_, even at lower concentrations, harmful effects of CO on COPD hospitalizations can be observed at the population level.

The exposure–response curves: In this study, the exposure–response curves of the effects of air PM_2.5_, PM_10_, SO_2_, NO_2_, and CO on COPD hospital admissions showed a significant positive relationship and were approximately linear without thresholds. However, for O_3-8h_, the exposure–response curve was not statistically significant. The results were similar to those of most studies on the exposure–response curve (10, 20, 56). Such as, a study in Lahore City, Pakistan (10) showed that the exposure–response curves of PM_2.5_ on COPD mortality were approximately linear with no indication of thresholds. A study in Ganzhou, China (20) also revealed a positive linear relationship between CO exposure and COPD hospitalization risk, and they did not observe any obvious threshold concentration below which CO has no effect or negative effect on the risk of COPD hospitalization. However, other studies have shown that the exposure–response curve between air pollutants and COPD is non-linear (23, 47). For instance, a time-stratified case-crossover study in Qingyang, China, analyzed the relationship between air pollutants and acute exacerbation of COPD hospitalizations, which found that the exposure–response curves were approximately linear for PM_2.5_ and that the curves for PM_10_, SO_2_, and CO were complicated, but only if the statistically significant part was considered. There was an associated “V” shape for PM_10_, a “U” shape for NO_2_, and an inverted “V” for SO_2_, CO, and O_3_. This may be related to the different concentrations of pollutants in different regions. Furthermore, different study designs (including different cities, population structures, inclusion, and exclusion criteria for study subjects), social institutions, and cultures may also account for the inconsistencies (20).

### Effect modifications by age

In this study, the harmful effect of PM on those aged <65 years was slightly higher than aged ≥65 years; however, the effect of gaseous pollutants on those aged <65 years was slightly lower than on those aged ≥65 years, which is inconsistent with other similar studies conducted in China (13, 20, 25, 47). A large population cohort study in southern China (25) reported that long-term PM exposure increased the risk of COPD mortality and that it seems to be more pronounced among older adult participants. An ecological study in Jinan (13) found that short-term exposure to NO_2_ and SO_2_ was associated with increased risks of daily COPD admissions, especially for the older adult. A time-stratified case-crossover study in Qingyang (47) showed that the harmful effects of PM_2.5_, PM_10_, SO_2_, NO_2_, and CO on acute exacerbation of COPD hospitalization were stronger in aged 15–64 years. While a time series study in Ganzhou (20) reported that there was no significant effect modification of age on CO exposure-associated hospitalizations for COPD. The older adult are more susceptible to the impact of air pollutants, which may be due to their normal pathological aging and weakened immune function; meanwhile, the older adult demographic usually suffers from preexisting chronic diseases and more advanced COPD (25). While people aged <65 years spend more time outdoors due to work, education, social interactions, and other reasons, which significantly increases their exposure to air pollutants (47). In addition, people aged <65 years are more likely to expose themselves to risk factors for respiratory diseases such as smoking and alcohol consumption, making them more susceptible to pollutants.

### Effect modifications by gender

In this study, the adverse effects of air pollutants on female COPD hospitalization were slightly higher than that on males. These findings were supported by existing evidence (13, 20, 25, 47). However, there are a few papers with inconsistent results: One study (57) found that the adverse effects of air pollutants on males were higher than on females and one study (58) showed that the effect of air pollutants on males and females was not significantly different. This inconsistency may be related to the differences in genetic, anatomical, and physiological characteristics of the different genders. Compared with males, female lungs, and trachea are smaller, and under the same pollution conditions, female lungs and tracheal tissue experience greater pressure. Furthermore, female lung immunity may be weaker (13, 25). On the other hand, it may also be related to gender differences in occupational distribution, personal characteristics (such as lifestyle, smoking, and other hobbies), or socioeconomic status: males are more engaged in industrial or taxi driving occupations or outdoor activities, have poor awareness of occupational protection, and have unhealthy habits such as smoking and drinking, making them more susceptible to the impact of air pollutants.

### Effect modifications by season

At present, there are few reports on the seasonal effects of air pollutants on COPD hospitalization. In this study, the influence of air pollutants on COPD in the cold season was greater than that in the warm season. This is consistent with Cheng’s (13) research findings in Jinan, but contrary to Song’s (20) findings (the CO exposure-associated hospitalization risk for COPD was stronger during the warm season). Our previous research also showed that the influence of air pollutants on respiratory diseases (19) and pneumonia (18) in the cold season was greater than that in warm seasons. This may be related to the higher concentration of pollutants in Lanzhou during the cold season.

In this study, the COPD hospital admissions data of 25 hospitals in Lanzhou City were used to analyze the impact of air pollutants on COPD hospitalizations. The lag effect and exposure–response curves were conducted, as well as a stratified analysis by age, gender, and season. However, the research still has some limitations. First, this is an ecological study. Although confounding factors such as temperature and humidity were adjusted during model fitting, there are still some potential factors (such as the use of air conditioners and purifiers) that may affect the relationship between air pollutants and COPD. At the same time, genetics, early-life events, respiratory infections, individual characteristics (inhalation of tobacco, drugs, and other combustible substances), and occupational exposure are also associated with COPD (7). However, these potential factors and individual characteristics cannot be obtained in an ecological study; hence, their impact on the relationship between air pollutants and COPD was not considered, which may reduce the accuracy of the conclusion. In the future, analytical epidemiological methods and molecular epidemiological methods can be applied to more accurately analyze the impact of air pollutants on COPD and explore its mechanism based on fully considering individual characteristics and potential factors. Second, this paper analyzed the effects of conventional air pollutants on COPD only in Lanzhou City. The spatial heterogeneity of air pollutants on health in multiple cities is worthy of further study. At the same time, the health effects of particulate matter components (such as copper, nickel, manganese, zinc, and other elements), different particle sizes (such as PM1 and PM4), and new pollutants (such as atmospheric microplastics) are more worthy of further study in the future.

## Conclusion

In summary, this study found that daily COPD hospitalization was stably positively associated with PM_2.5_, PM_10_, SO_2_, NO_2_, and CO, and the exposure–response curves of the effects of air pollutants (except O_3-8h_) on COPD hospital admissions were significant positive relationship and without thresholds; that is, the risk of hospitalization for COPD increased with the increase of pollutant concentration. There were lag effects; the greatest harmful effects of air pollutants (except O_3-8h_) on COPD admissions were all found at lag07. The adverse effects of air pollutants (except O_3-8h_) on females were slightly higher than that on males. Meanwhile, the adverse effects of air pollutants (except O_3-8h_) on COPD in cold seasons were greater than those in warm seasons. The harmful effect of PM on those aged <65 years was slightly higher than those aged ≥65 years; however, the effect of gaseous pollutants on those aged <65 years was slightly lower than those aged ≥65 years. The harmful effect of gaseous pollutants (except O_3-8h_) on COPD-hospitalized patients was stronger than that of PM. O_3-8h_ has a weak and unstable effect on COPD hospital admissions.

## Data availability statement

The raw data supporting the conclusions of this article will be made available by the authors, without undue reservation.

## Footnotes

^1^ http://data.cma.cn/

## References

[1] Renzi M Scortichini M Forastiere F de Donato F Michelozzi P Davoli M. A nationwide study of air pollution from particulate matter and daily hospitalizations for respiratory diseases in Italy. Sci Total Environ. (2022) 807:151034. doi: 10.1016/j.scitotenv.2021.151034, PMID: 34666080

[2] Qiu H Wang L Luo L Shen M. Shen: gaseous air pollutants and hospitalizations for mental disorders in 17 Chinese cities: association, morbidity burden and economic costs. Environ Res. (2022) 204:111928. doi: 10.1016/j.envres.2021.111928, PMID: 34437848

[3] Zhang Q Meng X Shi S Kan L Chen R Kan H. Overview of particulate air pollution and human health in China: evidence, challenges, and opportunities. Innovation. (2022) 3:100312. doi: 10.1016/j.xinn.2022.100312, PMID: 36160941 PMC9490194

[4] GBD Risk Factors Collaborators. Global burden of 87 risk factors in 204 countries and territories, 1990-2019: a systematic analysis for the global burden of disease study 2019. Lancet. (2020) 396:1223–49. doi: 10.1016/s0140-6736(20)30752-2, PMID: 33069327 PMC7566194

[5] GBD Risk Factor Collaborators. Global, regional, and national comparative risk assessment of 84 behavioural, environmental and occupational, and metabolic risks or clusters of risks for 195 countries and territories, 1990-2017: a systematic analysis for the global burden of disease study 2017. Lancet. (2018) 392:1923–94. doi: 10.1016/s0140-6736(18)32225-6, PMID: 30496105 PMC6227755

[6] Safiri S Carson-Chahhoud K Noori M Nejadghaderi SA Sullman MJM Heris JA. Burden of chronic obstructive pulmonary disease and its attributable risk factors in 204 countries and territories, 1990-2019: results from the global burden of disease study 2019. BMJ. (2022) 378:e069679–13. doi: 10.1136/bmj-2021-069679, PMID: 35896191 PMC9326843

[7] Stolz D Mkorombindo T Schumann DM Agusti A Ash SY Bafadhel M. Towards the elimination of chronic obstructive pulmonary disease: a lancet commission. Lancet. (2022) 400:921–72. doi: 10.1016/s0140-6736(22)01273-9, PMID: 36075255 PMC11260396

[8] Adeloye D Song P Zhu Y Campbell H Sheikh A Rudan I. Global, regional, and national prevalence of, and risk factors for, chronic obstructive pulmonary disease (COPD) in 2019: a systematic review and modelling analysis. Lancet Respir Med. (2022) 10:447–58. doi: 10.1016/s2213-2600(21)00511-7, PMID: 35279265 PMC9050565

[9] Sin DD Doiron D Agusti A Anzueto A Barnes PJ Celli BR. Air pollution and COPD: GOLD 2023 committee report. Eur Respir J. (2023) 61:2202469. doi: 10.1183/13993003.02469-2022, PMID: 36958741

[10] Malhi HM Ahmed I Nawaz R Nasir AH. Assessment of attributable proportion of particulate matter (PM_2.5_ and PM_10_) to different mortalities in Lahore city, Pakistan. Global NEST J. (2023) 25:84–90. doi: 10.30955/gnj.004422

[11] Wang X Chen L Cai M Tian F Zou H Qian Z. Air pollution associated with incidence and progression trajectory of chronic lung diseases: a population-based cohort study. Thorax. (2023) 78:698–705. doi: 10.1136/thorax-2022-219489, PMID: 36732083

[12] Brunekreef B Strak M Chen J Andersen ZJ Atkinson R Bauwelinck M. Mortality and morbidity effects of long-term exposure to low-level PM_2.5_, BC, NO_2_, and O_3_: an analysis of European cohorts in the ELAPSE project. Res Rep Health Eff Inst. (2021) 208:1–127. PMC9476567 36106702

[13] Cheng C Han C Fang Q Liu Y Chi X Li X. Associations between air pollutants and hospital admissions for chronic obstructive pulmonary disease in Jinan: potential benefits from air quality improvements. Environ Sci Pollut Res Int. (2023) 30:46435–45. doi: 10.1007/s11356-023-25567-8, PMID: 36717420 PMC9887246

[14] Ma M Chen Y Ding F Pu Z Liang X. The representativeness of air quality monitoring sites in the urban areas of a mountainous city. J Meteorol Res. (2019) 33:236–50. doi: 10.1007/s13351-019-8145-7

[15] Filonchyk M Yan H Li X. Temporal and spatial variation of particulate matter and its correlation with other criteria of air pollutants in Lanzhou, China, in spring-summer periods. Atmos Pollut Res. (2018) 9:1100–10. doi: 10.1016/j.apr.2018.04.011

[16] Bao H Dong J Liu X Tan E Shu J Li S. Association between ambient particulate matter and hospital outpatient visits for chronic obstructive pulmonary disease in Lanzhou, China. Environ Sci Pollut Res Int. (2020) 27:22843–54. doi: 10.1007/s11356-020-08797-y, PMID: 32323237

[17] Dong J You J Wang J Bao H. Association between short-term ambient air pollution and outpatient visits for acute exacerbation of chronic obstructive pulmonary disease in Lanzhou, 2013-19. Environ Geochem Health. (2022) 45:2495–509. doi: 10.1007/s10653-022-01363-0, PMID: 36006580

[18] Jin L Zhou T Fang S Zhou X Han B Bai Y. The short-term effects of air pollutants on pneumonia hospital admissions in Lanzhou, China, 2014–2019: evidence of ecological time-series study. Air Qual Atmos Health. (2022) 15:2199–213. doi: 10.1007/s11869-022-01244-6

[19] Jin L Zhou T Fang S Zhou X Bai Y. Association of air pollutants and hospital admissions for respiratory diseases in Lanzhou, China, 2014–2019. Environ Geochem Health. (2023) 45:941–59. doi: 10.1007/s10653-022-01256-2, PMID: 35384572 PMC8985563

[20] Song J Qiu W Huang X Guo Y Chen W Wang D. Association of ambient carbon monoxide exposure with hospitalization risk for respiratory diseases: a time series study in Ganzhou, China. Front Public Health. (2023) 11:1106336. doi: 10.3389/fpubh.2023.1106336, PMID: 36866098 PMC9972102

[21] Sofwan NM Mahiyuddin WRW Latif MT Ayub NA Yatim ANM Mohtar AAA. Risks of exposure to ambient air pollutants on the admission of respiratory and cardiovascular diseases in Kuala Lumpur. Sustain Cities Soc. (2021) 75:103390. doi: 10.1016/j.scs.2021.103390

[22] Ko FW Tam W Wong TW Lai CK Wong GW Leung TF. Effects of air pollution on asthma hospitalization rates in different age groups in Hong Kong. Clin Exp Allergy. (2007) 37:1312–9. doi: 10.1111/j.1365-2222.2007.02791.x, PMID: 17845411

[23] Huh J-Y Hong J Han D-W Park Y-J Jung J Lee SW. The impact of air pollutants and meteorological factors on chronic obstructive pulmonary disease exacerbations a Nationwide study. Ann Am Thorac Soc. (2022) 19:214–26. doi: 10.1513/AnnalsATS.202103-298OC, PMID: 34499589

[24] Dabrowiecki P Chcialowski A Dabrowiecka A Piorkowska A Badyda A. Air pollution and long-term risk of hospital admission due to chronic obstructive pulmonary disease exacerbations in Poland: a time-stratified, case-crossover study. Pol Arch Intern Med. (2023) 133:16444. doi: 10.20452/pamw.16444, PMID: 36856604

[25] Wang Y Du Z Zhang Y Chen S Lin S Hopke PK. Long-term exposure to particulate matter and COPD mortality: insights from causal inference methods based on a large population cohort in southern China. Sci Total Environ. (2023) 863:160808. doi: 10.1016/j.scitotenv.2022.160808, PMID: 36502970

[26] Ross BA Doiron D Benedetti A Aaron SD Chapman K Hernandez P. Short-term air pollution exposure and exacerbation events in mild to moderate COPD: a case-crossover study within the CanCOLD cohort. Thorax. (2023) 78:974–82. doi: 10.1136/thorax-2022-219619 37147124

[27] DeVries R Kriebel D Sama S. Outdoor air pollution and COPD-related emergency department visits, hospital admissions, and mortality: a Meta-analysis. COPD. (2017) 14:113–21. doi: 10.1080/15412555.2016.1216956, PMID: 27564008 PMC8994423

[28] Woo H Koehler K Putcha N Lorizio W McCormack M Peng R. Principal stratification analysis to determine health benefit of indoor AIR pollution reduction in a randomized environmental intervention in COPD: results from the CLEAN AIR study. Sci Total Environ. (2023) 868:161573. doi: 10.1016/j.scitotenv.2023.161573, PMID: 36669663 PMC9975085

[29] Wang Q Liu S. The effects and pathogenesis of PM_2.5_ and its components on chronic obstructive pulmonary disease. Int J Chron Obstruct Pulmon Dis. (2023) 18:493–506. doi: 10.2147/copd.s402122, PMID: 37056681 PMC10086390

[30] Choi J Shim JJ Lee MG Rhee CK Joo H Lee JH. Association between air pollution and viral infection in severe acute exacerbation of chronic obstructive pulmonary disease. J Korean Med Sci. (2023) 38:e68. doi: 10.3346/jkms.2023.38.e68, PMID: 36880109 PMC9988434

[31] Liu Y Yuan Q Zhang X Chen Z Jia X Wang M. Fine particulate matter (PM_2.5_) induces inhibitory memory alveolar macrophages through the AhR/IL-33 pathway. Cell Immunol. (2023) 386:104694. doi: 10.1016/j.cellimm.2023.104694, PMID: 36871457

[32] Paplinska-Goryca M Misiukiewicz-Stepien P Proboszcz M Nejman-Gryz P Gorska K Zajusz-Zubek E. Interactions of nasal epithelium with macrophages and dendritic cells variously alter urban PM-induced inflammation in healthy, asthma and COPD. Sci Rep. (2021) 11:13259. doi: 10.1038/s41598-021-92626-w, PMID: 34168212 PMC8225888

[33] Li R Kou X Tian J Meng Z Cai Z Cheng F. Effect of sulfur dioxide on inflammatory and immune regulation in asthmatic rats. Chemosphere. (2014) 112:296–304. doi: 10.1016/j.chemosphere.2014.04.065, PMID: 25048919

[34] Manisalidis I Stavropoulou E Stavropoulos A Bezirtzoglou E. Environmental and health impacts of ai pollution: a review. Front Public Health. (2020) 8:14. doi: 10.3389/fpubh.2020.00014, PMID: 32154200 PMC7044178

[35] Hendryx M Luo J Chojenta C Byles JE. Air pollution exposures from multiple point sources and risk of incident chronic obstructive pulmonary disease (COPD) and asthma. Environ Res. (2019) 179:108783. doi: 10.1016/j.envres.2019.108783, PMID: 31590000

[36] Wang Z Zhou Y Zhang Y Huang X Duan X Chen D. Association of change in air quality with hospital admission for acute exacerbation of chronic obstructive pulmonary disease in Guangdong, China: a province-wide ecological study. Ecotoxicol Environ Saf. (2021) 208:111590. doi: 10.1016/j.ecoenv.2020.111590, PMID: 33396113

[37] Wang SX Zhao B Cai SY Klimont Z Nielsen CP Morikawa T. Emission trends and mitigation options for air pollutants in East Asia. Atmos Chem Phys. (2014) 14:6571–603. doi: 10.5194/acp-14-6571-2014

[38] Wang Z Shi X Pan C Wang S. Spatial and temporal characteristics of environmental air quality and its relationship with seasonal climatic conditions in eastern China during 2015-2018. Int J Environ Res Public Health. (2021) 18:4524. doi: 10.3390/ijerph18094524, PMID: 33923225 PMC8123133

[39] Vahedian M Khanjani N Mirzaee M Koolivand A. Associations of short-term exposure to air pollution with respiratory hospital admissions in Arak, Iran. J Environ Health Sci Eng. (2017) 15:17. doi: 10.1186/s40201-017-0277-z, PMID: 28725443 PMC5514473

[40] Luo L Zhang Y Jiang J Luan H Yu C Nan P. Short-term effects of ambient air pollution on hospitalization for respiratory disease in Taiyuan, China: a time-series analysis. Int J Environ Res Public Health. (2018) 15:2160. doi: 10.3390/ijerph15102160, PMID: 30275384 PMC6210308

[41] Chang Q Zhang H Zhao Y. Ambient air pollution and daily hospital admissions for respiratory system-related diseases in a heavy polluted city in Northeast China. Environ Sci Pollut Res Int. (2020) 27:10055–64. doi: 10.1007/s11356-020-07678-8, PMID: 31933086

[42] Gao N. Short-term effects of ambient air pollution on admissions, lung function and systemic inflammation among chronic obstruetive pulmonary disease patients in Beijing, China. Beijing: Peking Union Med College. (2019). doi: 10.27648/d.cnki.gzxhu.2019.000178

[43] Cheng J Su H Xu Z. Intraday effects of outdoor air pollution on acute upper and lower respiratory infections in Australian children. Environ Pollut. (2021) 268:115698. doi: 10.1016/j.envpol.2020.115698, PMID: 33049483

[44] Bourbeau J Doiron D Biswas S Smith BM Benedetti A Brook JR. Associations with lung function and chronic obstructive pulmonary disease in the Canadian cohort obstructive lung disease study. Am J Respir Crit Care Med. (2022) 206:44–55. doi: 10.1164/rccm.202106-1439OC, PMID: 35380941 PMC9954329

[45] Aranburu-Imatz A Jimenez-Hornero JEE Morales-Cane I Lopez-Soto PJ. Environmental pollution in north-eastern Italy and its influence on chronic obstructive pulmonary disease: time series modelling and analysis using visibility graphs. Air Qual Atmos Health. (2023) 16:793–804. doi: 10.1007/s11869-023-01310-7, PMID: 36714016 PMC9875196

[46] Hoffmann C Maglakelidze M von Schneidemesser E Witt C Hoffmann P Butler T. Asthma and COPD exacerbation in relation to outdoor air pollution in the metropolitan area of Berlin, Germany. Respir Res. (2022) 23:64. doi: 10.1186/s12931-022-01983-1, PMID: 35307034 PMC8935815

[47] Liu Y Han X Cui X Zhao X Zhao X Zheng H. Association between air pollutants and acute exacerbation of chronic obstructive pulmonary disease: a time stratified case-crossover design with a distributed lag nonlinear model. Geohealth. (2022) 6:e2021GH000529. doi: 10.1029/2021GH000529, PMID: 35128294 PMC8802523

[48] United States Environment Protection Agency. Health effects of ozone pollution. (2022). Available at:https://www.epa.gov/ground-level-ozone-pollution/health-effects-ozone-pollution

[49] Gao N Li C Ji J Yang Y Wang S Tian X. Short-term effects of ambient air pollution on chronic obstructive pulmonary disease admissions in Beijing, China (2013-2017). Int J Chron Obstruct Pulmon Dis. (2019) 14:297–309. doi: 10.2147/COPD.S188900, PMID: 30774327 PMC6350834

[50] Qu F Liu F Zhang H Chao L Guan J Li R. The hospitalization attributable burden of acute exacerbations of chronic obstructive pulmonary disease due to ambient air pollution in Shijiazhuang, China. Environ Sci Pollut Res Int. (2019) 26:30866–75. doi: 10.1007/s11356-019-06244-1, PMID: 31446603

[51] Medina-Ramón M Zanobetti A Schwartz J. The effect of ozone and PM_10_ on hospital admissions for pneumonia and chronic obstructive pulmonary disease: a national multicity study. Am J Epidemiol. (2006) 163:579–88. doi: 10.1093/aje/kwj078, PMID: 16443803

[52] Jhun I Fann N Zanobetti A Hubbell B. Effect modification of ozone-related mortality risks by temperature in 97 US cities. Environ Int. (2014) 73:128–34. doi: 10.1016/j.envint.2014.07.009 25113626

[53] Danesh Yazdi M Wang Y Di Q Wei Y Requia WJ Shi L. Long-term association of air pollution and hospital admissions among medicare participants using a doubly robust additive model. Circulation. (2021) 143:1584–96. doi: 10.1161/circulationaha.120.050252, PMID: 33611922 PMC8055197

[54] Chen RJ Lee YH Chen TH Chen YY Yeh YL Chang CP. Carbon monoxide-triggered health effects: the important role of the inflammasome and its possible crosstalk with autophagy and exosomes. Arch Toxicol. (2021) 95:1141–59. doi: 10.1007/s00204-021-02976-7, PMID: 33554280

[55] Ierodiakonou D Zanobetti A Coull BA Melly S Postma DS Boezen HM. Ambient air pollution, lung function, and airway responsiveness in asthmatic children. J Allergy Clin Immunol. (2016) 137:390–9. doi: 10.1016/j.jaci.2015.05.028, PMID: 26187234 PMC4742428

[56] Hasegawa K Tsukahara T Nomiyama T. Short-term associations of low-level fine particulate matter (PM_2.5_) with cardiorespiratory hospitalizations in 139 Japanese cities. Ecotoxicol Environ Saf. (2023) 258:114961. doi: 10.1016/j.ecoenv.2023.114961, PMID: 37137261

[57] Priyankara S Senarathna M Jayaratne R Morawska L Abeysundara S Weerasooriya R. Ambient PM_2.5_ and PM_10_ exposure and respiratory disease hospitalization in Kandy, Sri Lanka. Int J Environ Res Public Health. (2021) 18:9617. doi: 10.3390/ijerph18189617, PMID: 34574538 PMC8466407

[58] Phosri A Ueda K Phung VLH Tawatsupa B Honda A Takano H. Effects of ambient air pollution on daily hospital admissions for respiratory and cardiovascular diseases in Bangkok, Thailand. Sci Total Environ. (2019) 651:1144–53. doi: 10.1016/j.scitotenv.2018.09.183, PMID: 30360246

