# Supplementary material for: The effect of air pollutants on COPD-hospitalized patients in Lanzhou, China (2015–2019)
Source: Front Public Health. 2024 Sep 19;12:1399662. doi: 10.3389/fpubh.2024.1399662 (PMC11446802; doi:10.3389/fpubh.2024.1399662)
Supplement: Supplementary file 1 [file Data_Sheet_1.docx]

**The effect of air pollutants on COPD in Lanzhou, China (2015-2019)**

**Contents**

Table S1. RR (95% CIs) of COPD hospital admissions with an increase of 10μg/m^3^ in air pollutants (1 mg/m^3^ in CO) in Lanzhou, China, 2015-2019

Table S2. RRs (95% CIs) of COPD hospital admissions with an increase of 10μg/m^3^ in air pollutants (1 mg/m^3^ in CO) in cold season

Table S3 RRs (95% CIs) of COPD hospital admissions with an increase of 10μg/m^3^ in air pollutants (1 mg/m^3^ in CO) in worm season

Table S4 Sensitivity analysis of association between COPD hospital admissions and air pollutants: controlling for different degrees of freedom (*df*) for time

Table S5 RR (95% CIs) of COPD hospital admissions with an increase of 10μg/m^3^ in air pollutants (1 mg/m^3^ in CO) from single-pollutant and two-pollutant models

Table S1. RR (95% CIs) of COPD hospital admissions with an increase of 10μg/m^3^ in air pollutants (and 1 mg/m^3^ in CO) in Lanzhou, China, 2015-2019

| Lag  days | PM_2.5_ | | | PM_10_ | | | SO_2_ | | | NO_2_ | | | O_3-8h_ | | | CO | | |
| --- | --- | --- | --- | --- | --- | --- | --- | --- | --- | --- | --- | --- | --- | --- | --- | --- | --- | --- |
|  | RR | 95% CI | | RR | 95% CI | | RR | 95% CI | | RR | 95% CI | | RR | 95% CI | | RR | 95% CI | |
| lag0 | **1.013** | **(1.001,** | **1.025)** | 1.003 | (1.000, | 1.006) | 1.002 | (0.973, | 1.032) | 1.008 | (0.992, | 1.025) | 1.012 | (0.996, | 1.028) | 1.028 | (0.976, | 1.082) |
| lag1 | 1.002 | (0.989, | 1.014) | 1.000 | (0.997, | 1.003) | 1.011 | (0.980, | 1.043) | 1.002 | (0.985, | 1.020) | 0.996 | (0.980, | 1.012) | 1.009 | (0.954, | 1.067) |
| lag2 | 1.007 | (0.998, | 1.015) | 1.002 | (1.000, | 1.004) | 1.010 | (0.988, | 1.032) | 1.009 | (0.998, | 1.021) | 1.001 | (0.988, | 1.014) | 1.012 | (0.975, | 1.050) |
| lag3 | 1.005 | (1.000, | 1.011) | 1.001 | (1.000, | 1.002) | 1.005 | (0.991, | 1.020) | 1.006 | (0.998, | 1.014) | 1.006 | (0.997, | 1.015) | 1.015 | (0.991, | 1.039) |
| lag4 | 1.003 | (0.998, | 1.009) | 1.000 | (0.999, | 1.001) | 1.005 | (0.990, | 1.019) | 1.002 | (0.994, | 1.010) | 1.006 | (0.997, | 1.016) | 1.018 | (0.993, | 1.042) |
| lag5 | 1.004 | (0.998, | 1.009) | 1.000 | (0.999, | 1.001) | 1.009 | (0.996, | 1.023) | 1.002 | (0.994, | 1.009) | 1.001 | (0.993, | 1.010) | 1.020 | (0.997, | 1.044) |
| lag6 | **1.005** | **(1.001,** | **1.009)** | 1.001 | (1.000, | 1.002) | **1.018** | **(1.008,** | **1.028)** | 1.004 | (0.999, | 1.010) | 0.993 | (0.987, | 1.000) | **1.023** | **(1.007,** | **1.040)** |
| lag7 | **1.008** | **(1.000,** | **1.017)** | 1.002 | (1.000, | 1.004) | **1.028** | **(1.007,** | **1.050)** | 1.008 | (0.997, | 1.020) | **0.983** | **(0.970,** | **0.996)** | 1.026 | (0.991, | 1.063) |
| lag01 | **1.015** | **(1.003,** | **1.027)** | 1.003 | (1.000, | 1.006) | 1.013 | (0.981, | 1.046) | 1.011 | (0.994, | 1.028) | 1.008 | (0.989, | 1.027) | 1.037 | (0.984, | 1.093) |
| lag02 | **1.022** | **(1.009,** | **1.034)** | **1.005** | **(1.002,** | **1.008)** | 1.023 | (0.990, | 1.056) | **1.020** | **(1.004,** | **1.038)** | 1.009 | (0.989, | 1.029) | 1.049 | (0.996, | 1.104) |
| lag03 | **1.027** | **(1.013,** | **1.042)** | **1.006** | **(1.002,** | **1.009)** | 1.028 | (0.992, | 1.064) | **1.026** | **(1.007,** | **1.046)** | 1.015 | (0.993, | 1.037) | **1.064** | **(1.005,** | **1.127)** |
| lag04 | **1.031** | **(1.016,** | **1.046)** | **1.006** | **(1.002,** | **1.009)** | 1.032 | (0.997, | 1.070) | **1.028** | **(1.009,** | **1.049)** | 1.021 | (0.999, | 1.043) | **1.083** | **(1.022,** | **1.148)** |
| lag05 | **1.034** | **(1.018,** | **1.051)** | **1.006** | **(1.002,** | **1.010)** | **1.042** | **(1.003,** | **1.082)** | **1.030** | **(1.008,** | **1.052)** | **1.023** | **(1.001,** | **1.045)** | **1.105** | **(1.038,** | **1.177)** |
| lag06 | **1.040** | **(1.023,** | **1.057)** | **1.007** | **(1.002,** | **1.011)** | **1.061** | **(1.021,** | **1.102)** | **1.035** | **(1.012,** | **1.058)** | 1.015 | (0.995, | 1.036) | **1.131** | **(1.060,** | **1.207)** |
| lag07 | **1.048** | **(1.030,** | **1.067)** | **1.008** | **(1.004,** | **1.013)** | **1.091** | **(1.048,** | **1.135)** | **1.043** | **(1.018,** | **1.068)** | 0.998 | (0.981, | 1.016) | **1.160** | **(1.084,** | **1.242)** |

Table S2. RRs (95% CIs) of COPD hospital admissions with an increase of 10μg/m^3^ in air pollutants (1 mg/m^3^ in CO) in cold season

| Lag  days | PM_2.5_ | | PM_10_ | | SO_2_ | | NO_2_ | | O_3-8h_ | | CO | |
| --- | --- | --- | --- | --- | --- | --- | --- | --- | --- | --- | --- | --- |
|  | RR | 95% CI | RR | 95% CI | RR | 95% CI | RR | 95% CI | RR | 95% CI | RR | 95% CI |
| lag0 | 1.011 | (0.993,1.029) | 1.003 | (0.999,1.007) | 0.981 | (0.942,1.022) | 1.001 | (0.977,1.026) | 1.018 | (0.994,1.043) | 1.021 | (0.955,1.091) |
| lag1 | 1.006 | (0.986,1.026) | 1.001 | (0.996,1.005) | 1.024 | (0.982,1.067) | 1.015 | (0.989,1.041) | 1.004 | (0.980,1.030) | 1.021 | (0.949,1.098) |
| lag2 | 1.009 | (0.995,1.023) | 1.002 | (0.999,1.005) | 1.011 | (0.982,1.041) | 1.018 | (1.000,1.036) | 1.008 | (0.988,1.029) | 1.031 | (0.982,1.082) |
| lag3 | 1.007 | (0.998,1.016) | 1.000 | (0.998,1.002) | 1.008 | (0.989,1.028) | 1.008 | (0.996,1.020) | 1.008 | (0.994,1.021) | 1.033 | (1.000,1.066) |
| lag4 | 1.005 | (0.997,1.014) | 1.000 | (0.997,1.002) | 1.011 | (0.991,1.031) | 1.000 | (0.988,1.012) | 1.003 | (0.988,1.018) | 1.031 | (0.998,1.065) |
| lag5 | 1.006 | (0.997,1.014) | 1.000 | (0.998,1.002) | 1.013 | (0.994,1.032) | 0.999 | (0.988,1.011) | 0.995 | (0.982,1.009) | 1.027 | (0.996,1.059) |
| lag6 | **1.008** | **(1.002,1.013)** | **1.001** | **(1.000,1.003)** | **1.016** | **(1.002,1.030)** | 1.003 | (0.995,1.011) | **0.986** | **(0.975,0.997)** | **1.023** | **(1.001,1.046)** |
| lag7 | 1.010 | (0.998,1.023) | **1.003** | **(1.000,1.006)** | 1.018 | (0.990,1.047) | 1.009 | (0.992,1.026) | **0.976** | **(0.955,0.997)** | 1.018 | (0.974,1.065) |
| lag01 | 1.017 | (0.998,1.037) | **1.004** | **(1.000,1.008)** | 1.005 | (0.958,1.054) | 1.016 | (0.987,1.045) | 1.022 | (0.992,1.053) | 1.042 | (0.965,1.125) |
| lag02 | **1.026** | **(1.008,1.045)** | **1.005** | **(1.001,1.010)** | 1.016 | (0.968,1.066) | **1.034** | **(1.005,1.064)** | 1.031 | (0.998,1.064) | 1.074 | (0.995,1.159) |
| lag03 | **1.033** | **(1.013,1.055)** | **1.006** | **(1.001,1.010)** | 1.025 | (0.973,1.078) | **1.042** | **(1.010,1.075)** | **1.038** | **(1.003,1.075)** | **1.109** | **(1.021,1.204)** |
| lag04 | **1.039** | **(1.017,1.061)** | **1.005** | **(1.000,1.010)** | 1.036 | (0.983,1.091) | **1.042** | **(1.008,1.077)** | **1.041** | **(1.005,1.079)** | **1.143** | **(1.050,1.244)** |
| lag05 | **1.044** | **(1.02,1.069)** | **1.005** | **(1.000,1.011)** | 1.049 | (0.992,1.110) | **1.041** | **(1.003,1.081)** | 1.036 | (0.999,1.075) | **1.174** | **(1.070,1.287)** |
| lag06 | **1.052** | **(1.027,1.078)** | **1.007** | **(1.001,1.013)** | **1.066** | **(1.006,1.129)** | **1.044** | **(1.004,1.086)** | 1.022 | (0.988,1.057) | **1.201** | **(1.091,1.321)** |
| lag07 | **1.063** | **(1.036,1.091)** | **1.010** | **(1.003,1.017)** | **1.085** | **(1.022,1.152)** | **1.054** | **(1.011,1.098)** | 0.997 | (0.966,1.029) | **1.222** | **(1.108,1.348)** |

Table S3. RRs (95% CIs) of COPD hospital admissions with an increase of 10μg/m^3^ in air pollutants (1 mg/m^3^ in CO) in warm season

| Lag  days | PM_2.5_ | | PM_10_ | | SO_2_ | | NO_2_ | | O_3_ | | CO | |
| --- | --- | --- | --- | --- | --- | --- | --- | --- | --- | --- | --- | --- |
|  | RR | 95% CI | RR | 95% CI | RR | 95% CI | RR | 95% CI | RR | 95% CI | RR | 95% CI |
| lag0 | 1.001 | (0.982,1.020) | 1.001 | (0.997,1.005) | 1.003 | (0.939,1.071) | 0.995 | (0.965,1.027) | 1.005 | (0.984,1.026) | 0.927 | (0.795,1.082) |
| lag1 | 0.996 | (0.980,1.013) | 0.998 | (0.994,1.002) | 1.020 | (0.956,1.088) | 0.999 | (0.968,1.030) | 0.994 | (0.973,1.015) | 1.055 | (0.904,1.232) |
| lag2 | 1.003 | (0.990,1.016) | 1.000 | (0.997,1.003) | 1.034 | (0.984,1.087) | 1.009 | (0.987,1.031) | 0.996 | (0.979,1.013) | 1.001 | (0.892,1.123) |
| lag3 | 1.003 | (0.994,1.011) | 1.000 | (0.998,1.002) | 1.005 | (0.972,1.039) | 1.009 | (0.994,1.024) | 1.005 | (0.994,1.016) | 1.023 | (0.943,1.111) |
| lag4 | 1.000 | (0.991,1.009) | 0.999 | (0.997,1.002) | 0.980 | (0.946,1.016) | 1.005 | (0.989,1.021) | 1.009 | (0.997,1.021) | 1.040 | (0.955,1.133) |
| lag5 | 0.997 | (0.989,1.006) | 0.999 | (0.997,1.001) | 0.976 | (0.944,1.009) | 1.000 | (0.986,1.015) | 1.006 | (0.995,1.017) | 1.013 | (0.936,1.095) |
| lag6 | 0.995 | (0.987,1.002) | 0.999 | (0.997,1.001) | 0.985 | (0.960,1.011) | 0.995 | (0.985,1.006) | 0.997 | (0.989,1.005) | 0.958 | (0.900,1.019) |
| lag7 | 0.992 | (0.979,1.006) | 0.999 | (0.996,1.002) | 1.002 | (0.956,1.051) | 0.991 | (0.971,1.011) | 0.985 | (0.969,1.002) | 0.892 | (0.796,1.000) |
| lag01 | 0.997 | (0.978,1.017) | 0.999 | (0.994,1.004) | 1.023 | (0.939,1.115) | 0.994 | (0.956,1.033) | 0.998 | (0.974,1.023) | 0.979 | (0.812,1.180) |
| lag02 | 1.000 | (0.978,1.023) | 0.999 | (0.994,1.005) | 1.058 | (0.965,1.160) | 1.003 | (0.962,1.046) | 0.994 | (0.970,1.020) | 0.980 | (0.799,1.201) |
| lag03 | 1.002 | (0.977,1.029) | 1.000 | (0.993,1.006) | 1.064 | (0.963,1.175) | 1.012 | (0.967,1.058) | 0.999 | (0.973,1.027) | 1.003 | (0.804,1.250) |
| lag04 | 1.002 | (0.975,1.030) | 0.999 | (0.992,1.005) | 1.043 | (0.941,1.156) | 1.017 | (0.971,1.064) | 1.009 | (0.982,1.036) | 1.043 | (0.833,1.305) |
| lag05 | 0.999 | (0.970,1.029) | 0.998 | (0.991,1.005) | 1.018 | (0.911,1.137) | 1.017 | (0.969,1.068) | 1.014 | (0.987,1.042) | 1.056 | (0.836,1.334) |
| lag06 | 0.994 | (0.964,1.025) | 0.997 | (0.989,1.004) | 1.003 | (0.894,1.125) | 1.012 | (0.963,1.064) | 1.011 | (0.986,1.037) | 1.011 | (0.804,1.273) |
| lag07 | 0.986 | (0.953,1.020) | 0.996 | (0.988,1.004) | 1.005 | (0.891,1.133) | 1.003 | (0.953,1.055) | 0.996 | (0.974,1.019) | 0.903 | (0.721,1.129) |

Table S4 Sensitivity analysis of association between COPD hospital admissions and air pollutants: controlling for different degrees of freedom (*df*) for time ^a^

|  | PM_2.5_ | PM_10_ | SO_2_ | NO_2_ | O_3-8h_ | CO |
| --- | --- | --- | --- | --- | --- | --- |
| *df*=6 | 1.045(1.027,1.064) | 1.008(1.003,1.013) | 1.107(1.066,1.150) | 1.05(1.026,1.075) | 1.025(1.003,1.047) | 1.186(1.110,1.267) |
| *df*=7 | 1.048(1.030,1.067) | 1.008(1.004,1.013) | 1.091(1.048,1.135) | 1.043(1.018,1.068) | 1.023(1.001,1.045) | 1.160(1.084,1.242) |
| *df*=8 | 1.042(1.022,1.062) | 1.006(1.001,1.011) | 1.095(1.054,1.138) | 1.048(1.024,1.073) | 1.014(0.992,1.037) | 1.161(1.085,1.243) |
| *df*=9 | 1.039(1.019,1.059) | 1.006(1.001,1.011) | 1.068(1.023,1.115) | 1.036(1.011,1.062) | 1.013(0.991,1.035) | 1.124(1.046,1.207) |
| *df*=10 | 1.038(1.019,1.059) | 1.006(1.001,1.011) | 1.067(1.022,1.115) | 1.036(1.010,1.063) | 1.012(0.990,1.035) | 1.130(1.051,1.216) |

***Note.*** ^a^ Results obtained from single-pollutant models for lag07 day, except O_3-8h_ for lag05 day.

Table S5 RR (95% CIs) of COPD hospital admissions with an increase of 10μg/m^3^ in air pollutants (1 mg/m^3^ in CO) from single-pollutant and two-pollutant models

| Model | PM_2.5_ | PM_10_ | SO_2_ | NO_2_ | O_3-8h_ | CO |
| --- | --- | --- | --- | --- | --- | --- |
| Without adjustment | 1.048(1.030,1.067) | 1.008(1.004,1.013) | 1.091(1.048,1.135) | 1.043(1.018,1.068) | 1.023(1.001,1.045) | 1.160(1.084,1.242) |
| Adjusted for PM_2.5_ | — | — | 1.047(0.999,1.096) | 1.022(0.996,1.048) | 1.012(0.990,1.034) | 1.069(0.983,1.163) |
| Adjusted for PM_10_ | — | — | 1.084(1.030,1.141) | 1.048(1.017,1.081) | 1.015(0.988,1.043) | 1.145(1.050,1.249) |
| Adjusted for SO_2_ | 1.036(1.014,1.058) | 1.006(1.000,1.011) | — | 1.022(1.017,1.051) | 1.021(0.999,1.044) | 1.089(0.953,1.245) |
| Adjusted for NO_2_ | 1.038(1.014,1.063) | 1.007(1.000,1.013) | 1.070(1.030,1.123) |  | 1.024(0.988,1.046) | 1.126(1.050,1.225) |
| Adjusted for O_3-8h_ | 1.046(1.027,1.065) | 1.008(1.003,1.013) | 1.082(1.037,1.129) | 1.038(1.008,1.069) |  | 1.146(1.067,1.230) |
| Adjusted for CO | 1.036(1.014,1.060) | 1.006(1.001,1.011) | 1.047(0.967,1.132) | 1.017(0.988,1.048) | 1.021(0.999,1.044) | — |
